# Assessment in Work Productivity and the Relationship with Cognitive Symptoms (AtWoRC): primary analysis from a Canadian open-label study of vortioxetine in patients with major depressive disorder (MDD)

**DOI:** 10.1017/S1092852918000913

**Published:** 2019-06

**Authors:** Pratap Chokka, Joanna Bougie, Emmanouil Rampakakis, Jean Proulx

**Affiliations:** 1 Grey Nuns Community Hospital, Edmonton, Alberta, Canada; 2 Lundbeck Canada Inc., Montreal, Quebec, Canada; 3 JSS Medical Research, Montreal, Quebec, Canada

**Keywords:** Cognitive dysfunction, functional impairment, major depressive disorder, real life, vortioxetine, work productivity

## Abstract

**Objective:**

The Assessment in Work Productivity and the Relationship with Cognitive Symptoms (AtWoRC) study aimed to assess the association between cognitive symptoms and work productivity in gainfully employed patients receiving vortioxetine for a major depressive episode (MDE).

**Methods:**

Patients diagnosed with major depressive disorder (MDD) and treated with vortioxetine independently of study enrollment were assessed over 52 weeks at visits that emulated a real-life setting. Patients were classified as those receiving vortioxetine as the first treatment for their current MDE (first treatment) or having shown inadequate response to a previous antidepressant (switch). The primary endpoint was the correlation between changes in patient-reported cognitive symptoms (20-item Perceived Deficits Questionnaire [PDQ-D-20]) and changes in work productivity loss (Work Limitations Questionnaire [WLQ]) at week 12. Additional assessments included changes in symptom and disease severity, cognitive performance, functioning, work loss, and safety.

**Results:**

In the week 12 primary analysis, 196 eligible patients at 26 Canadian sites were enrolled, received at least one treatment dose, and attended at least one postbaseline study visit. This analysis demonstrated a significant, strong correlation between PDQ-D-20 and WLQ productivity loss scores (*r*=0.634; *p*<0.001), and this correlation was significant in both first treatment and switch patients (*p*<0.001). A weaker correlation between Digit Symbol Substitution Test and WLQ scores was found (*r*=−0.244; *p*=0.003).

**Conclusion:**

At 12 weeks, improvements in cognitive dysfunction were significantly associated with improvements in workplace productivity in patients with MDD, suggesting a role for vortioxetine in functional recovery in MDD.

## Introduction

Major depressive disorder (MDD) is a highly prevalent, burdensome condition that affects more than 350 million people worldwide.^1^^,^^2^ In Canada, the prevalence of MDD was found to be 3.9% in 2012, and it is higher in women and younger age groups.αDD is a multidimensional disease that requires assessment and treatment of various aspects, which include emotional, physical, and cognitive symptoms.^4^ There are more than 1000 combinations of symptoms per the *Diagnostic and Statistical Manual of Mental Disorders*, 5th ed. (DSM-V), indicating that MDD is a very heterogeneous disease.^5^

Cognitive dysfunction is a core feature of depression, which is ultimately associated with functional impairment and work limitations.^6^^–^^8^ In fact, the World Health Organization (WHO) considers depression to be the leading cause of disability worldwide.^9^ Based on previous studies, the estimated prevalence of unemployment and disability in patients with MDD range from 18% to 34%, and the absentee rates range from 23% to 53% across Canadian provinces.^10^ In Canada, depression costs more than US$9 billion due to lost productivity (i.e., absence from work and attending work while unwell), and depression-related presenteeism (i.e., reduced productivity at work due to MDD) costs US$6.8 billion,^11^ demonstrating that MDD is associated with significant economic burden.^12^

The clinical relevance of cognitive dysfunction in MDD and its role in work-related disability is supported by a large body of evidence.^6^^,^^13^^–^^20^ For the management of adults with MDD, the Canadian Network for Mood and Anxiety Treatments released clinical guidelines in 2016, which state that recovery from depression involves both relief of symptoms and improvement of functioning.^4^^,^^21^ However, systematic reviews have shown that improvement in mood symptoms is only modestly correlated with functional outcomes, and few studies of antidepressants have assessed functional outcomes.^15^^,^^18^^,^^22^^–^^26^

Previously, the landmark STAR*D study, which highlighted the impact of individual depressive symptoms on functional impairment, identified sad mood, concentration, and fatigue as the top three symptoms that have a high relative impact on functional change.^27^ Cognition has been reported to mediate a quarter of the impact of a major depressive episode (MDE) on work loss,^18^ and cognitive symptoms have been associated with greater workplace impairment than depression severity.^8^ Previous studies have reported that greater functional disability and impairment in daily activities were associated with more severe depression and greater perceived cognitive dysfunction, while patients with more severe perceived cognitive dysfunction reported worse work-related productivity outcomes.^28^ Therefore, treatment of cognitive symptoms may hold the key to meaningful functional improvement, yet there is a lack of understanding of the relationship between cognitive dysfunction and functional impairment in MDD.^29^

Vortioxetine is a multimodal antidepressant that is indicated for the treatment of MDD in adults. It differs from selective serotonin reuptake inhibitors and serotonin norepinephrine reuptake inhibitors due to its direct effect on 5-hydroxytryptamine (5-HT) receptors, including 5-HT_1A_ receptor agonism, 5-HT_1B_ receptor partial agonism, and 5-HT_3_, 5-HT_1D_, and 5-HT_7_ receptor antagonism.^30^

Previous studies have shown that vortioxetine is effective for the treatment of MDD.^31^^,^^32^ In addition, studies have shown that vortioxetine is able to improve the cognitive symptoms of MDD. For example, post hoc analyses of a randomized trial in adult patients with MDD who were treated with vortioxetine demonstrated an improvement in the Digital Symbol Substitution Test (DSST), which is an objective cognitive measure of attention/speed of processing, executive functioning, and memory.^33^ The FOCUS study also showed similar improvement in DSST scores in patients treated with vortioxetine.^34^ Moreover, the post hoc analysis from the FOCUS study demonstrated that greater improvement in the measures of cognitive functioning was observed in the working versus total population treated with vortioxetine.^35^ Furthermore, in the CONNECT study, adult patients with recurrent MDD who were treated with vortioxetine had a significant improvement in functional capacity (assessed by the UCSD Performance-Based Skills Assessment [UPSA]) compared with placebo. This study also showed that vortioxetine was superior to placebo in improving the Work Limitations Questionnaire (WLQ) Time Management score, indicating that there may be a relationship between the improvement of cognitive symptoms and the improvement of work productivity with vortioxetine.^36^

Given the established efficacy profile of vortioxetine in improving mood symptoms as well as cognitive symptoms, and the potential relationship between the improvement of cognitive symptoms and work productivity, the Assessment in Work Productivity and the Relationship with Cognitive Symptoms (AtWoRC) study examined the association between cognitive dysfunction and workplace productivity in employed/student patients with MDD treated with vortioxetine. This paper reports results from the 12-week analysis, including analysis of the primary endpoint.

## Methods

### Study design

**Figure 1:**
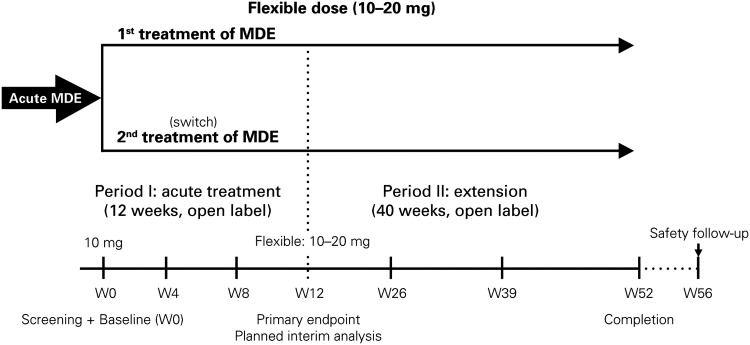
Study design of the AtWoRC study. MDE=major depression episode

The AtWoRC study (NCT02332954) is an interventional, open-label, single-cohort study. It was conducted at 26 sites across Canada by 9 psychiatrists and 17 primary-care physicians. To emulate as closely as possible a real-life setting, structured investigator-administered interventions and interviews were minimized. The research was approved by the local ethics boards of participating academic sites and the Institutional Review Board Services (Aurora, ON, Canada). Vortioxetine was given at a flexible, open-label dosing of 10–20 mg daily per the Canadian Product Monograph.^37^ Primary endpoint was evaluated at week 12 (analysis after all recruited patients attended the week 12 visit), with follow-ups scheduled every 4 weeks. Patients will then have a follow-up at week 52 (study completion), and a final safety follow-up at week 56 (Figure 1). Data from the final week 52 analysis and from the safety follow-up will be reported elsewhere, as the study is ongoing.

Patients were stratified by treatment history: (1) patients who received vortioxetine as the first treatment for their current MDE (first treatment); and (2) patients who had inadequate response to a previous antidepressant at labeled doses for at least 6 weeks, and it was the opinion of the investigator that treatment with another antidepressant was warranted (switch/second treatment).

In this study, the Pearson correlation coefficient was the primary statistical parameter used to assess the association between cognitive dysfunction and workplace productivity in patients. Given that this is a single-cohort study, the sample size calculations were driven by the required precision of the correlation coefficient estimate obtained in the study. Precision was assessed with the 95% confidence interval (CI) of the point estimate (i.e., 95% CI width of ±20% of the point estimate). For the outcome measures to be clinically important, a correlation coefficient of ≥0.50 would be required. Therefore, assuming a correlation coefficient of 0.50 and a required 95% CI of ±0.10 (20% of the point estimate), the total sample size of the study was estimated to be 200 patients.

### Assessment tools

The assessment tools used in the study included patient-rated and clinician-rated measures. The patient-rated tools included the Perceived Deficits Questionnaire for Depression (PDQ-D-20), the WLQ productivity loss, the Quick Inventory of Depressive Symptomatology–Self-Report (QIDS-SR), the 12-item WHO Disability Assessment Schedule 2.0 (WHODAS 2.0) questionnaire, the Work Productivity and Activity Impairment (WPAI) questionnaire, the Sheehan Disability Scale (SDS), and the Generalized Anxiety Disorder 7-item (GAD-7) questionnaire. The clinician-rated tools included the Clinical Global Impression–Severity (CGI-S) and CGI–Improvement (CGI-I) scales. The assessment tools also include the DSST (a neuropsychological test). All tools are described in Supplementary Table 1.

### Inclusion and exclusion criteria

The main inclusion criteria required patients to be between 18 and 65 years of age. A patient’s employment status had to be gainfully employed (working ≥20 hours/week) or enrolled full-time in postsecondary studies or vocational training. The diagnosis of MDD needed to be confirmed according to the DSM-V, and current MDE was confirmed by the investigator, with reported duration of the current MDE of at least 3 months. Patients must have had a baseline score of ≥15 in the QIDS-SR assessment and a baseline score of ≥30 in the PDQ-D-20 assessment.

The main exclusion criteria included a score of >69 on the DSST at screening/baseline, to preserve assay sensitivity; current diagnosis or history of manic or hypomanic episode, schizophrenia, or any other psychotic disorder, including major depression with psychotic features; personality disorder, mental retardation, pervasive development disorder, attention-deficit hyperactivity disorder, organic mental disorders, or mental disorders due to a general medical condition (DSM-V criteria); physical, cognitive, or language impairment of such severity as to adversely affect the validity of the data derived from the patient-reported outcomes; current depressive symptoms that were considered by the investigator to have been resistant to two adequate antidepressant treatments of at least 6 weeks duration, each at the maximum recommended dose according to Canadian labeling; and previous exposure to vortioxetine.

### Study endpoints

The primary endpoint of this study was to describe the correlation between change from baseline to week 12 in patient-reported cognitive symptoms (assessed by PDQ-D-20) and work productivity loss (assessed by WLQ) in gainfully employed patients receiving vortioxetine for an MDE. Baseline depression severity was controlled for in the primary analysis.

The secondary endpoints were as follows: change in cognitive symptoms and performance (PDQ-D-20 and DSST); change in symptom and disease severity (QIDS-SR, CGI-I, and CGI-S); change in functioning and work productivity (WLQ productivity loss, SDS, WPAI, and WHODAS 2.0); treatment response rate, where response was defined as a change in QIDS-SR of ≥50% from baseline; and remission rate, which was defined as having a QIDS-SR total score of ≤5. Pharmacoeconomic parameters (i.e., work loss) of the whole cohort were described.

Safety and tolerability of vortioxetine were assessed with the incidence of adverse events (AEs), which were coded according to the MedDRA dictionary of terms and were described with the proportion of patients with one or more events within each system organ class and preferred term.

### Statistical Analysis

The study used the following analysis sets: all patients treated set (APTS), which included all patients with a valid baseline assessment who took at least one dose of vortioxetine; and full analysis set (FAS), which included all patients from the APTS who had at least one complete postbaseline study visit.

For the primary endpoint, the correlation between the change from baseline to week 12 in the PDQ-D-20 score and the change from baseline to week 12 in the WLQ productivity loss score was described by the partial correlation coefficient conditional on age, sex, baseline PDQ-D-20, baseline WLQ, disease duration, and disease severity. The analyses were conducted for the 12-week data (first treatment group, switch group, and the total population). To evaluate the impact of missing data at 12 weeks, additional sensitivity analysis was done using the last observation carried forward (LOCF) method.

For the secondary endpoints, the following descriptive statistics were used for the changes from baseline to week 12 in various scores: mean, median, standard deviation (*SD*), and 95% CIs of the mean. The rates of response to treatment and remission were described as the proportion of patients achieving these endpoints at week 12 and the corresponding 95% CIs. Differences between first treatment and switch patients with respect to the baseline and week 12, and changes from baseline to week 12 in the study outcome parameters were described with the Student *t* test for independent samples. For functioning and work productivity measures (WLQ, SDS, WPAI, and WHODAS 2.0 scores) and treatment response, all correlations were described with the Pearson correlation and the intraclass correlation coefficient. The secondary correlations (Pearson correlations) were “cross-sectional” at week 12.

## Results

### Patient disposition and baseline clinical characteristics

**Figure 2:**
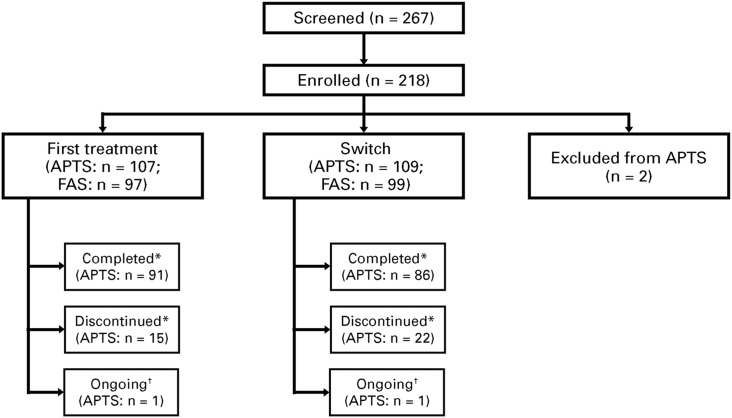
Patient disposition. *“Completed” refers to those who attended the Week 12 visit. “Discontinued” refers to treatment discontinuation before Week 12. †Ongoing analysis. APTS=all patients treated set; FAS=full analysis set

As of November 2016, 218 patients were enrolled at 26 sites, with 216 eligible patients (*n*=107 for first treatment, *n*=109 for switch; 2 patients were excluded) who received at least one treatment dose, and formed the APTS. From these patients, 196 patients (*n*=97 for first treatment, *n*=99 for switch) attended at least one postbaseline study visit and formed the FAS (Figure 2).

At baseline, switch patients were significantly older than those in the first treatment group (38.9 years in first treatment group vs. 42.7 years in switch group; *p*=0.024; Table 1) and had longer disease duration (*p*<0.001). Over 90% of patients in both groups were gainfully employed or independent. The mean doses of vortioxetine (*SD*) at week 12 were 14.6 mg (5.0), 15.8 mg (5.1), and 15.1 mg (5.1) in the first treatment group, switch group, and total population, respectively.

**Table 1:** Baseline patient demographics and clinical characteristics*

	First treatment (*n*=107)	Switch (*n*=109)	Total (APTS: *n*=216)
Mean age, years (*SD*)†	38.9 (12.7)	42.7 (12.0)	40.8 (12.4)
Female gender, % (*n*)	69.2 (74)	69.7 (76)	69.4 (150)
Caucasian race, % (*n*)	92.5 (99)	95.4 (104)	94.0 (203)
Mean duration of disease, years (*SD*)†	5.9 (6.9)	10.8 (10.9)	8.4 (9.4)
Employment type, % (*n*)			
Employed/independent	89.7 (87)	93.9 (93)	91.8 (180)
Full-time vocational	4.1 (4)	1.0 (1)	2.6 (5)
Full-time postsecondary student	6.2 (6)	3.0 (3)	4.6 (9)
Clinical characteristics: mean scores at baseline (week 0), mean (*SD*)			
	First treatment (*n*=97)	Switch (*n*=99)	Total (FAS: *n*=196)
PDQ-D-20	49.7 (12.1)	49.5 (12.1)	49.6 (12.0)
QIDS-SR	18.7 (2.6)	18.1 (2.6)	18.4 (2.6)
GAD-7†	15.5 (4.7)	14.0 (4.8)	14.8 (4.8)
CGI-S	4.1 (0.6)	4.1 (0.5)	4.1 (0.5)
WLQ, % productivity loss	13.0 (4.8)	13.7 (4.3)	13.4 (4.6)
WPAI, % overall impairment	66.0 (23.7)	69.1 (22.7)	67.6 (23.2)
SDS	21.0 (4.8)	21.0 (5.5)	21.0 (5.1)
WHODAS 2.0	21.1 (6.8)	21.0 (7.9)	21.0 (7.4)
DSST (correct symbols)	47.5 (11.2)	45.0 (12.1)	46.2 (11.7)

* For patient demographics, patients in the APTS were assessed. For clinical characteristics, patients in the FAS were assessed.

† Significantly different between groups.

APTS=all patients treated set; CGI-S=Clinical Global Impression–Severity; DSST=Digit Symbol Substitution Test; GAD-7=Generalized Anxiety Disorder 7-item scale; FAS=full analysis set; PDQ-D-20=Perceived Deficits Questionnaire for Depression; QIDS–SR=Quick Inventory of Depressive Symptomatology–Self-Report; SDS=Sheehan Disability Scale; WHODAS 2.0=12-item World Health Organization Disability Assessment Schedule 2.0; WLQ=Work Limitations Questionnaire; WPAI=Work Productivity and Activity Impairment.

As shown in Table 1, the mean (*SD*) PDQ-D-20 scores at baseline were 49.7 (12.1) and 49.5 (12.1) in the first treatment and switch groups, respectively. The mean (*SD*) WLQ productivity loss was 13.0% (4.8) and 13.7% (4.3) in the first treatment and switch groups, respectively (the maximum attainable score for the WLQ productivity loss is 25%). Overall, patients had severe cognitive dysfunction, severe depression, severe anxiety, and functional impairment. The baseline disease severity was similar in both groups, with the exception of the level of anxiety (GAD-7), which was greater in the first treatment group (15.5 in first treatment vs. 14.0 in switch; *p*=0.034).

### Primary endpoint

In terms of the association between changes from baseline to week 12 in PDQ and WLQ productivity loss, there was a statistically significant, strong correlation between PDQ-D-20 and WLQ scores in the FAS (*r*=0.634; *p*<0.001; Table 2). The results indicate that patients who had improved cognitive function following treatment with vortioxetine also had improved workplace productivity. The correlation between PDQ-D-20 scores and WLQ productivity loss was also significant in both first treatment patients (*r*=0.679; *p*<0.001) and switch patients (*r*=0.577; *p*<0.001). To evaluate the impact of missing data at week 12, additional sensitivity analysis was performed using the LOCF method. The results were similar to those from the observed case analysis (FAS: *r*=0.638; *p*<0.001; first treatment: *r*=0.675; *p*<0.001; switch: *r*=0.609; *p*<0.001). At week 12, the largest changes from baseline in the WLQ productivity loss were observed in the time management, mental-interpersonal demands, and output demands domains (results not shown).

**Table 2:** Partial correlation between change in PDQ-D-20 and change in WLQ productivity loss from baseline to week 12* (OC)

Group	*n*	*r*	*p*-value
OC			
FAS	151	0.634	<0.001
First treatment	79	0.679	<0.001
Switch	72	0.577	<0.001

* Controlled for age, sex, baseline PDQ-D-20, baseline WLQ productivity loss, disease duration, and disease severity (baseline QIDS-SR, baseline CGI-S).

CGI-S=Clinical Global Impression–Severity; FAS=full analysis set; OC=observed cases; PDQ-D-20=Perceived Deficits Questionnaire for Depression; QIDS-SR=Quick Inventory of Depressive Symptomatology–Self-Report; WLQ=Work Limitations Questionnaire.

### Secondary endpoints

At week 12, patients treated with vortioxetine showed significant improvement from baseline according to all assessment scores, including measures of cognitive symptoms, disease severity, functional outcomes, work productivity, disability, and objective cognitive performance (Table 3). In addition, the change in DSST was weakly correlated with the change in WLQ productivity loss in the FAS (Table 4). When this analysis is repeated with the removal of the covariates, the correlation becomes statistically significant, although still weak (*r*=−0.244; *p*=0.003; Table 5). The associations between all outcome parameters at week 12 (i.e., the actual test scores) are shown in Supplementary Table 2.

**Table 3:** Mean change in different test scores from baseline to week 12 in the FAS (OC)*

Mean (*SD*)	First treatment (*n*=97)	Switch (*n*=99)	Total (FAS) (*n*=196)
PDQ-D-20	−23.7 (17.3)	−25.5 (15.8)	−24.6 (16.6)
QIDS-SR	−9.8 (5.3)	−10.4 (5.0)	−10.1 (5.2)
CGI-S	−1.2 (1.1)	−1.1 (1.0)	−1.1 (1.1)
WLQ, % productivity loss	−6.1 (6.3)	−7.1 (6.1)	−6.6 (6.2)
WPAI, % overall impairment	−31.3 (34.6)	−34.3 (32.4)	−32.7 (33.5)
SDS	−10.2 (9.0)	−10.8 (8.9)	−10.5 (8.9)
WHODAS	−9.9 (9.8)	−10.1 (8.9)	−10.0 (9.3)
DSST	7.8 (11.9)	10.1 (13.8)	8.9 (12.9)

* All changes are *p*<0.0001 (paired *t* test) compared with baseline.

CGI-S=Clinical Global Impression–Severity; DSST=Digit-Symbol Substitution Test; FAS=full analysis set; OC=observed cases; PDQ-D-20=Perceived Deficits Questionnaire for Depression; QIDS-SR=Quick Inventory of Depressive Symptomatology–Self-Report; SDS=Sheehan Disability Scale; WHODAS 2.0=12-item World Health Organization Disability Assessment Schedule 2.0; WLQ=Work Limitations Questionnaire; WPAI=Work Productivity and Activity Impairment.

**Table 4:** Partial correlation between change in DSST and change in WLQ productivity loss from baseline to week 12 in the FAS (OC)

Group	*r*	*p*-value
Total (FAS)	−0.172	0.040
First treatment	−0.255	0.032
Switch	−0.084	0.513

* Controlled for age, sex, baseline DSST, baseline WLQ productivity loss, disease duration, and disease severity (baseline QIDS-SR, baseline CGI-S).

CGI-S=Clinical Global Impression–Severity; DSST=Digit Symbol Substitution Test; FAS=full analysis set; OC=observed cases; QIDS-SR=Quick Inventory of Depressive Symptomatology–Self-Report; WLQ=Work Limitations Questionnaire.

**Table 5:** Pearson correlation between change in DSST and change in WLQ productivity loss from baseline to week 12 in the FAS

	First treatment		Switch		FAS	
	*r*	*p*-value*	*r*	*p*-value*	*r*	*p*-value*
Week 12	−0.227	0.045	−0.248	0.037	−0.244	0.003

* *p*-value was calculated using *t* test for testing of Pearson’s correlation coefficient.

DSST=Digit Symbol Substitution Test; FAS=full analysis set; WLQ=Work Limitations Questionnaire.

**Figure 3:**
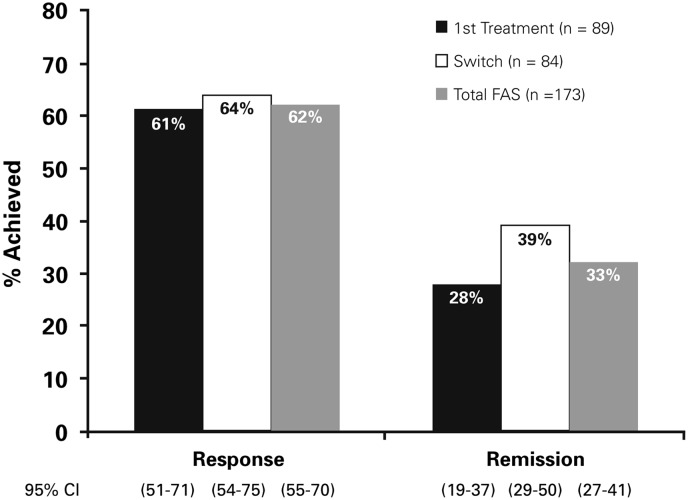
Response and remission rates at Week 12 (OC). FAS=Full Analysis Set; OC=observed cases

The treatment response rates at week 12 were 61% (95% CI, 51%–71%) and 64% (95% CI, 54%–75%) in the first treatment and switch groups, respectively (Figure 3). There were 28% (95% CI, 19%–37%) and 39% (95% CI, 29%–50%) of first treatment and switch patients, respectively, who achieved remission at week 12.

In terms of work loss at 12 weeks, treatment with vortioxetine resulted in fewer patients reporting missing work due to depression compared with baseline within the previous 3 months (57% at baseline vs. 22% at week 12). In patients who reported missing work at baseline and week 12 visits, fewer missed work days were reported within the previous 3 months at the week 12 visit compared with baseline (12 days at baseline vs. 7 days at week 12; Supplementary Figure 1).

### Safety

The safety profile and tolerability of vortioxetine were consistent with the Canadian product monograph without any new safety signals observed. The most common AEs (frequency ≥5% in APTS, *n*=216) were nausea, headache, insomnia, drug ineffectiveness, and nasopharyngitis (Table 6). Withdrawal of consent was the main reason for study discontinuation (6.5% of total patient population), and there were 5 patients (4.7%) in the first treatment group and 5 patients (4.6%) in the switch group who discontinued treatment due to an AE. Other common reasons for treatment discontinuation are outlined in Supplementary Table 3.

**Table 6:** Most common AEs in APTS

Event	% patients (*n*)
Nausea	27.8 (60)
Headache	12.5 (27)
Insomnia	9.7 (21)
Drug ineffective	7.4 (16)
Nasopharyngitis	6.9 (15)

AE=adverse event; APTS=all patients treated set.

## Discussion

At present, there are many studies investigating MDD and response to antidepressant treatment in the clinical setting, but the working MDD patient population has not been well studied in a real-life setting. Therefore, it is important to gather practical data on this disorder such that clinical evidence can be translated into the real world and the true benefits of antidepressant treatments in routine clinical care can be demonstrated. Cognitive dysfunction in MDD has been reported to play a role in work productivity impairment. The 12-week analysis of the AtWoRC study demonstrated that improvements in cognitive function were strongly correlated with improvements in workplace productivity in gainfully employed patients with MDD receiving vortioxetine in a real-life setting.

As shown in Table 2, changes in PDQ-D-20 scores were found to be significantly correlated with WLQ productivity loss scores. These results suggest that treatment of perceived cognitive symptoms with vortioxetine may increase workplace productivity. Given that returning to optimal levels of gainful employment is an essential element of patient re-integration, and given that the patients in this study improved globally on all investigated measures, including those assessing cognitive symptoms, these results suggest that vortioxetine is an effective option.

After 12 weeks of treatment with vortioxetine, patients showed significant improvements in perceived cognitive symptoms and cognitive performance (as shown by PDQ-D-20 and DSST, respectively), symptoms and disease severity (as shown by QIDS, CGI-S), work productivity (as shown by WLQ productivity loss and WPAI), and functioning (as shown by SDS and WHODAS 2.0; Table 3). The improvement in objective cognitive performance as assessed by the DSST and perceived cognitive dysfunction as assessed by the PDQ-D-20 were consistent with previous studies with vortioxetine.^31^^,^^32^^,^^34^^,^^36^ Moreover, results from the AtWoRC study demonstrated the efficacy of vortioxetine in working patients, which supported previous findings from the post hoc analysis of the FOCUS study that showed a significant improvement in the DSST scores with vortioxetine at 10 and 20 mg in the working patients (*p*<0.001). The analysis also showed that vortioxetine had a greater effect in working patients when compared with the total study population; a similar pattern was observed for the PDQ and Montgomery–Åsberg Depression Rating Scale scores.^35^ In addition, in the CONNECT study, vortioxetine was superior to placebo in the improvement of WLQ–Time Management score, indicating that there may be a correlation between improvement of cognitive symptoms and improvement in work productivity. Indeed, this relationship was examined and demonstrated in the AtWoRC study. Overall, the large-scale, placebo-controlled studies showed that patients treated with vortioxetine have consistent improvement in cognitive function. The present study reinforces these results in a real-life setting while highlighting the association between cognitive symptoms and work productivity.

Comparing with previous studies with vortioxetine, the response and remission rates were high, which were consistent with previous studies with vortioxetine.^30^^,^^38^^,^^39^

In the past, very few studies have explored the association between the change in perceived cognitive symptoms and change in workplace productivity. A previous report from the Combined Medications to Enhance Depression Outcomes (CO-MED) trial demonstrated this association by assessing perceived cognitive symptoms with QIDS–Clinician Rated and QIDS-SR, and work productivity with WPAI and the Massachusetts General Hospital Cognitive and Physical Functioning Questionnaire.^40^ The CO-MED trial also showed that patients with improved self-reported work productivity had 3 to 5 times greater remission rates at 3 months posttreatment. The AtWoRC study is one of the few studies that explored the association in a Canadian real-life setting, and it further validates and consolidates previous findings, including those from the CO-MED trial.

Despite the correlation between changes in subjective cognitive symptoms (PDQ-D-20) and work productivity (WLQ productivity loss) found in this study, only a weak correlation between changes in objective cognitive performance (DSST) and work functioning (WLQ productivity loss) was observed for the total patient population in the FAS. If the covariates were removed, there was a slightly stronger and statistically significant correlation between changes in DSST and WLQ productivity loss scores (Table 5), though the correlation was still weak. From these results, it is speculated that there are differences between subjective- and objective-rated cognitive assessment tools. In the study by Srisurapanont *et al*., discrepancy was found between subjective and objective measures of cognition in patients with MDD, and the authors concluded that age and depression severity might predict the discrepancy between the two sets of measures.^41^ When Lam *et al*. examined the effect of desvenlafaxine on neurocognitive and work functioning in working patients with MDD, they showed similar findings, in that there were no significant correlations between changes in objective cognitive function and changes in measures of work functioning.^42^ In another study, subjective and objective measures of cognition were also reported not to be associated with each other.^43^ Subjective cognitive impairment, but not objective cognitive impairment, was found to be significantly associated with psychosocial impairment; this difference might be attributed to differences in depression severity.

While the pharmacoeconomic parameters were descriptive in nature, improvements were indeed observed in the number of patients who missed work days and in the number of work days missed from baseline to week 12. This finding supports the primary endpoint result in that treatment with vortioxetine may also lead to reduced presenteeism in the workplace. Accordingly, this result highlights the importance of treating MDD in order to reduce its economic burden in addition to its clinical burden.

Overall, the safety profile of vortioxetine was consistent with previous studies and the product monograph. Common AEs included nausea, headache, and diarrhea, demonstrating that vortioxetine was well tolerated in the patient population. For first treatment and switch groups, the rate of discontinuation due to adverse drug reaction at week 12 was low (4.7% and 4.6%, respectively) compared with other open-label studies (11.3% with duloxetine as first treatment^44^; 6.3% when switched to duloxetine^45^). The most common reason for discontinuation in all groups was withdrawal of consent. There were 5 patients in the first treatment group and 5 patients in the switch group who discontinued treatment due to loss of follow-up. It is speculated that since patients in the AtWoRC study were mostly gainfully employed, study assessments might add burden to their well-being, which could lead to discontinuation of treatment.

There were a few limitations to this study, which included its open-label nature and potential placebo effect. However, it should be noted that the primary objective of the study was to evaluate the correlation between improvement in perceived cognitive symptoms and work productivity, and treatment effectiveness was assessed secondarily. A control group or a comparative treatment was also lacking. Hence, the improvements in the test scores observed in this study could not be directly attributed to a study or drug effect.

## Conclusions

In this 12-week analysis of the AtWoRC study, patients with depression who were treated with vortioxetine had improvements in cognitive function and improved workplace productivity. After 12 weeks of treatment with vortioxetine, both first treatment and switch patients showed significant improvements in cognitive symptoms and performance, disease severity, work productivity, and functioning; the results are consistent with previous studies with vortioxetine. The real-life data from the AtWoRC study confirmed that vortioxetine is an efficacious, safe, and well-tolerated treatment option for patients with MDD. Future results from the 52-week final analysis follow-up are expected later in 2018.

## Supplementary material

For supplementary material accompanying this paper visit https://doi.org/10.1017/S1092852918000913. click here to view supplementary material
